# Zoonotic *Giardia duodenalis* assemblage A in northern sloth from Brazilian Amazon

**DOI:** 10.1590/0074-02760230088

**Published:** 2023-11-13

**Authors:** Lisiane Lappe dos Reis, Lirna Salvioni Silva de Souza, Francisco Carlos de Oliveira Braga, Dayane Costa de Souza Lima, Natália Aparecida de Souza Lima, Jessica da Silva Padinha, Alessandra Ferreira Dales Nava, Ana Carolina Paulo Vicente

**Affiliations:** 1 Fundação Oswaldo Cruz-Fiocruz, Instituto Leônidas & Maria Deane, Laboratório de Diversidade Microbiana da Amazônia de Importância para a Saúde, Manaus, AM, Brasil; 2 Fundação Oswaldo Cruz-Fiocruz, Instituto Oswaldo Cruz, Programa de Pós-Graduação em Biologia Parasitária, Rio de Janeiro, RJ, Brasil; 3 Superintendência do Ibama no Amazonas, Centro de Triagem de Animais Silvestres, Manaus, AM, Brasil; 4 Fundação Oswaldo Cruz-Fiocruz, Instituto Leônidas & Maria Deane, Laboratório de Ecologia de Doenças Transmissíveis na Amazônia, Manaus, AM, Brasil; 5 Fundação Oswaldo Cruz-Fiocruz, Instituto Oswaldo Cruz, Laboratório de Genética Molecular de Microrganismos, Rio de Janeiro, RJ, Brasil

**Keywords:** Giardia duodenalis, assemblage, genotyping, sloth, Amazon, Bradypus tridactylus

## Abstract

**BACKGROUND:**

The parasite *Giardia duodenalis* infects a wide range of vertebrate hosts, including domestic and wild animals as well as humans. *Giardia* is genotyped into eight assemblages (A-H). Zoonotic assemblages A and B have already been identified in humans and wild and domestic animals (non-human primates and cats) from Brazilian Amazon and in the world. Due to its zoonotic/zooanthroponotic nature, surveillance initiatives and the definition of *Giardia* assemblages are important in order to characterise the epidemiological scenario and to implement further control measures.

**OBJECTIVES:**

Determine assemblages of *G. duodenalis* in sloths from the Brazilian Amazon Region.

**METHODS:**

Faecal parasitological examination of sloths from Amazonas State. Polymerase chain reaction (PCR) targeting the beta giardin (BG), and genes from multilocus sequence typing (MLST) scheme, amplicon sequencing and phylogenetic analysis.

**FINDINGS:**

Here, we identified, by microscopy, *Giardia* in two northern sloths (*Bradypus tridactylus*). These two samples were submitted to molecular assays and it was revealed that both were infected by *G. duodenalis* assemblage A. Phylogenetic analysis showed that they belong to assemblage A within sequences from humans and wild and domestic animals.

**CONCLUSION:**

Therefore, besides showing, by the first time, the current presence of this parasite in sloths, our findings reveals that this wild animal species would be part of the zoonotic/zooanthroponotic scenario of this parasite in the Brazilian Amazon.

*Giardia duodenalis* (syn. *G. intestinalis*, *G. lamblia*) is the flagellated protozoan that infects the small intestine of humans and others mammals in worldwide.^1^

Giardiasis is a disease associate with this parasitic infection in human, with symptoms such as acute diarrhoea which may progress to a chronic stage, but most infections remain asymptomatic.^2^ In children, giardiasis has a negative impact on their growth and cognitive development.^3^ In animals, cats and dogs, *G. duodenalis* infection can be associated with a spectrum of signs ranging from subclinical forms to acute or chronic diarrhoea.^4^ ^,^ ^5^ Overall, this waterborne and foodborne parasite is a major problem in one health, particularly, in areas with poor sanitary conditions.

Currently, *G. duodenalis* is classified into eight distinct assemblages or genotypes (A to H), where A and B infect humans and animals worldwide. While assemblages C-H show specificity to particular animal hosts: assemblages C and D occur in canines, assemblage E in ungulates including livestock, assemblage F in cats, assemblage G in rodents, and assemblage H is found in marine mammals, such as pinnipeds family.^6^ However, recently, changes were observed in this scenario since assemblages C, D, E, F and G were characterised in human infections.^(^ ^7^ ^,^ ^8^ ^,^ ^9^ ^,^ ^10^ ^)^

The Amazon Region is hot spot of biodiversity in the world and, so far, in the Brazilian Amazon, only assemblages A and B were identified in humans, non-human primates and cats, and assemblage C in dogs.^11^ ^,^ ^12^ Therefore, we have maintained a surveillance program of *Giardia* parasite identification particularly, considering wild animals from Brazilian Amazon Region. In this context, we reported the identification and characterisation of *G. duodenalis* assemblage A in two northern sloths (*Bradypus tridactylus)* by means of the beta giardin (BG), the caffeine-induced death protein-1 like protein (CID1) and mitotic control protein Dis3 (DIS3) genes analysis.

## MATERIALS AND METHODS

We performed parasitological examination by spontaneous sedimentation^13^ in single faecal specimens of the 27 sloths of the species *B. tridactylus* (12/27), and *Choloepus didactylus* (15/27) from Wild Animal Rehabilitation Centre (Cetas)/Ibama located in Manaus municipality (3º4’25”S, 60º0’20”W), in Amazonas State, Brazil, between January and May 2022. The presence of *Giardia* was confirmed by microscope observation. The sloths did not have gastrointestinal symptoms.

DNA was extracted from *Giardia* positive samples using QIAamp DNA Stool Mini Kit with minor modifications: lysis buffer temperature to 95ºC for 15 min, and 200 uL of elution buffer for 10 min at room temperature. Polymerase chain reaction (PCR) was carried out targeting the genes encoding BG,^14^ and from MLST scheme.^15^ We were successfully in the amplification the BG, CID1 and DIS3 genes. The amplicons were purified using PureLink Quick PCR Purification Kit (Invitrogen, Lithuania), according to the manufacturer’s instructions. The fragments were Sanger sequenced using BigDye Terminator Cycle Sequencing Ready Reaction Kit.

The nucleotide sequence was edited in BioEdit software and consensus sequence was aligned in MAFFT^16^ and alignment corrected in MEGAX software.

Phylogenetic analyses were conducted using the program implemented in Phylosuite:^(^ ^17^ ^,^ ^18^ ^)^ IQ-Tree v1.6.8^19^ was used for the Maximum likelihood (ML) analysis. The best-fit substitution models were selected with ModelFinder according to the corrected Akaike information criterion (AICc). The ML analysis was conducted with the TN+F+G4 model for the BG tree, and, for the concatenated tree with BG, CID1 and DIS3 alignments, the partition models selected were GTR+F+I (BG gene) and K2P+I (CID1 and DIS3 genes). The sequences used in the concatenated tree are in the Supplementary data (Table). The clade support was estimated using 5,000 replicates for both ultrafast bootstrap (UFBoot) and Shimodaira-Hasegawa approximate likelihood ratio test (SH-aLRT). Finally, two trees were built and visualised by Figtree v1.4.0 and further edited in Inkscape. The phylogenetic analyses were performed with sequences from humans and animals belonging to *G. duodenalis* assemblages A-F from worldwide and *G. muris* species.

This study was approved by the local SISBIO n.º 67153-3 (general license for animal collection), and by UFAM CEUA n.º 017/2020 (Federal University of Amazonas State Ethics Committee for Animal Use).

## RESULTS AND DISCUSSION

**Fig. 1:**
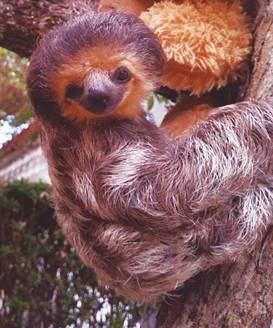
northern sloth (*Bradyprus tridactylus*) positive to *Giardia duodenalis* assemblage A.

**Fig. 2:**
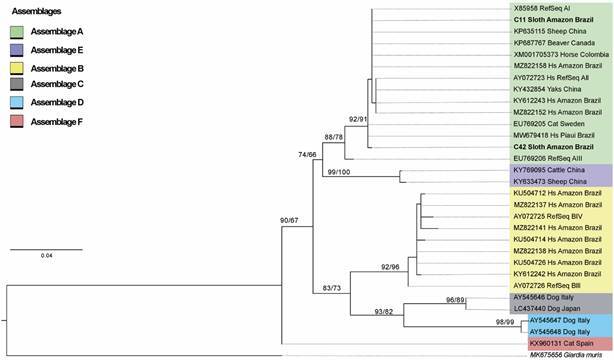
maximum-likelihood phylogenetic tree based on *Giardia duodenalis* beta giardin (BG) (466 bp). Support values for the clades UFBoot and Shimodaira-Hasegawa approximate likelihood ratio test (SH-aLRT) are presented at the left of the nodes. Both BG sloth sequences are in bold. Hs: *Homo sapiens*.

**Fig. 3:**
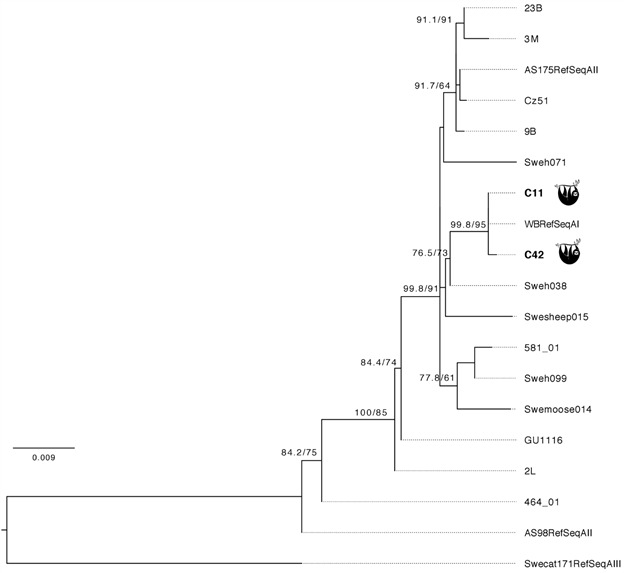
maximum-likelihood phylogenetic tree based on concatenated *Giardia duodenalis* beta giardin (BG), CID1 and DIS3 sequences (1750 bp). Support values for the clades UFBoot and Shimodaira-Hasegawa approximate likelihood ratio test (SH-aLRT) are presented at the left of the nodes. Both sloth sequences are label.

This study identified firstly, by microscopy observation, that 2/27 sloths of the species *B. tridactylus* were positive for *Giardia* (low parasitaemia). Both were baby sloths rescued in urban area of the Manaus municipality, Amazonas State, Brazil and delivered to Cetas/Ibama (Fig. 1). Curiously, studies around the world enrolling young animals demonstrate higher *Giardia* prevalence rates in the young than in adults.^20^ The BG, CID1 and DIS3 genes were successfully amplified in these two samples and their sequences confirm *G. duodenalis*. In addition, they were used to perform a phylogenetic analysis (Accession numbers: OQ971403, OQ971404, OR453867, OR453868, OR453869, OR453870). Based on the available BG sequences of the assemblages A, B, C, D and F from animals and humans from Amazon Region and worldwide, besides the sequence of *G. muris* species, a genetic tree was built up. This tree showed several clusters corresponding to *G. duodenalis* assemblages A-F and the BG gene sequences from both sloths clustered into two distinct sub-clusters of assemblage A (Fig. 2) within *G. duodenalis* from humans, wild and domestic animals around the world. Another tree was built using BG, CID1 and DIS3 sequences concatenated since the CID1 and DIS3 sequences available in Gen Bank are from assemblage A and, therefore, this tree represents only this assemblage (Fig. 3).

Assemblage A was recently identified in humans, cats and non-human primates from Manaus, Amazonas State,^11^ the same area were both sloths were found. Moreover, this genotype has been found in humans, domestic and wild animals from the Amazon Region,^(^ ^12^ ^,^ ^21^ ^)^ as well as in cosmopolitan regions around the world.^(^ ^7^ ^,^ ^22^ ^)^

Six species of sloths exist today: *C. hoffmanni* and *C. didactylus* are two-toed sloth, two of the most commonly kept species in zoologic societies; and the three-toed sloth, *B. torquatus*, *B. variegatus* (brown throated three-toed sloth), and *B. tridactylus* (pale-throated three-toed sloth). The latter species also called northern sloth, is restricted to the Guyana shield, and in Brazil it is only found north of the Amazon River and east of Negro River.^23^ This species has no endangered status, and its having been recorded as locally relatively abundant, although this species is threatened by the loss of forests and illegal pet trade.^24^

In 1928, *Giardia* spp. was identified in *B. variegatus* sloth captured in Panama and maintained in a zoo (USA), showing no clinical signs.^25^ Since then, there is no literature record about species and genotypes of *Giardia* in sloths in the world. Here, we identified, for the first time, the zoonotic assemblage A in sloths (*B. tridactylus*) from Manaus, Amazon State, Brazil.

Interestingly, *G. duodenalis* was identified, by immunodiagnosisin, in *Nothrotherium maquinense* (ground sloth) coprolites from the northeastern Brazilian Megafauna corresponding the late Pleistocene period, correlated to human occupation.^26^ The authors speculated that *Giardia* could be circulating among humans and megafauna and, since them, it has been adapted to several other species.

Therefore, our findings as well the previous evidences of *Giardia* in sloth species show that this wild mammal is part of the zoonotic/zooanthponotic scenario of this parasite in Brazilian Amazon.
