# Supplementary material for: Zoonotic Giardia duodenalis assemblage A in northern sloth from Brazilian Amazon
Source: Mem Inst Oswaldo Cruz. 2023 Nov 13;118:e230088. doi: 10.1590/0074-02760230088 (PMC10644951; doi:10.1590/0074-02760230088)
Supplement: Supplementary file 1 [file 1678-8060-mioc-118-e230088-s.pdf]

TABLE

Isolate ID and accession numbers of all sequences used in concatenated tree described in Ankarklev et al.[1] (grey), Woschke et al.[2] (blue), Franzen et al.[3] (green), Ankarklev et al.[4] (light green), Lebbad et al.[5] (yellow), Bonhomme et al.[6] (orange), Klotz et al.[7] (pink) and the present study (red)

Sequence code	Bg Isolate/acc nº	CID1 Isolate/acc nº	DIS3 Isolate/acc nº
1	WB FJ560591	WB MG520215	WB MG520263
2	AS98 CVLA00000000.1	AS98 MG520217	AS98 MG520265
3	AS175 CAHQ00000000.1	AS175 MG520219	AS175 MG520269
4	Swecat171 EU769206	Swecat171 MG520224	Swecat171 MG520270
5	Swesheep015 JF773747	Swesheep015 MG520222	Sweh038 MG520268
6	464-01 MT879082	Sweh038 MG520216	464-01 MT879093
7	C11 OQ971403	GU1116 MG520218	Cz51 OP450948
8	C42 OQ971404	Sweh099 MG520220	C11 OR453869
9		Sweh071 MG520221	C42 OR453870
10		Swemoose014 MG520223	
11		581-01 OP450944	
12		C11 OR453867	
13		C42 OR453868	

REFERENCES

1. Ankarklev J, Lebbad M, Einarsson E, Franzén O, Ahola H, Troell K, et al. A novel high-resolution multilocus sequence typing of *Giardia intestinalis* assemblage A isolates reveals zoonotic transmission, clonal outbreaks and recombination. Infect Genet Evol. 2018; 60: 7-16.

2. Woschke A, Faber M, Stark K, Holtfreter M, Mockenhaupt F, Richter J, et al. Suitability of current typing procedures to identify epidemiologically linked human *Giardia duodenalis* isolates. PLoS Negl Trop Dis. 2021; 15(3): e0009277.

3. Franzen O, Jerlstrom-Hultqvist J, Einarsson E, Ankarklev J, Ferella M, Andersson B, et al. Transcriptome profiling of *Giardia intestinalis* using strand-specific RNA-seq. PLoS Comput Biol. 2013; 9(3): E1003000.

4. Ankarklev J, Franzén O, Peirasmaki D, Jerlström-Hultqvist J, Lebbad M, Andersson J, et al. Comparative genomic analyses of freshly isolated *Giardia intestinalis* assemblage A isolates. BMC Genomics. 2015; 16(1): 1-14.

5. Lebbad M, Mattsson JG, Christensson B, Ljungström B, Backhans A, Andersson JO, et al. From mouse to moose: multilocus genotyping of *Giardia* isolates from various animal species. Vet Parasitol. 2010; 168(3-4): 231-9.

6. Bonhomme J, Le Goff L, Lemée V, Gargala G, Ballet JJ, Favennec L. Limitations of tpi and bg genes sub-genotyping for characterization of human *Giardia duodenalis* isolates. Parasitol Int. 2011; 60(3): 327-30.

7. Klotz C, Sannella AR, Weisz F, Chaudhry U, Sroka J, Tůmová P, et al. Extensive testing of a multi-locus sequence typing scheme for *Giardia duodenalis* assemblage A confirms its good discriminatory power. Parasit Vectors. 2022; 15(1): 489.
